# Association of Viral Load in SARS-CoV-2 Patients With Age and Gender

**DOI:** 10.3389/fmed.2021.608215

**Published:** 2021-01-27

**Authors:** Waleed H. Mahallawi, Ali Dakhilallah Alsamiri, Alaa Faisal Dabbour, Hamdah Alsaeedi, Abdulmohsen H. Al-Zalabani

**Affiliations:** ^1^Clinical Laboratory Technology, Taibah University, Madinah, Saudi Arabia; ^2^Regional Lab in Madinah, Ministry of Health, Madinah, Saudi Arabia; ^3^Department of Laboratories and Blood Banks, Ministry of Health, Madinah, Saudi Arabia; ^4^Clinical Laboratory Department, College of Applied Medical Sciences, Shaqra University, Shaqraa, Saudi Arabia; ^5^Department of Family and Community Medicine, College of Medicine, Taibah University, Madinah, Saudi Arabia

**Keywords:** SARS-CoV-2, age, gender, CT value, HESN

## Abstract

**Background:** The coronavirus disease 2019 (COVID-19), caused by the novel severe acute respiratory syndrome coronavirus-2 (SARS-CoV-2), is a global public health emergency. Age and sex are two important factors associated with risks and outcomes of various diseases. COVID-19 morbidity also seems to be affected by patient age and sex. It has been found that older age groups have more severe COVID-19 symptoms and higher fatality rates while children tend to have lower prevalence and milder symptoms than adults.

**Methods:** The study reviewed electronic medical records of COVID-19 patients from Madinah city, Saudi Arabia. The study included all cases who tested positive (*n* = 3,006) between March 20 and May 22, 2020. Data were obtained from the Health Electronic Surveillance Network (HESN) database.

**Results:** Approximately 80% of the study sample were males and half were in the 30–40-year-old age group. The Ct value of the whole sample ranged from 15.08 to 35, with a mean of 27.44 (SD: 5.23; 95% C.I. = 27.25–27.66). The means of Ct values varied between age groups from 27.05 to 27.82. Analysis of the mean differences between age groups using one-way ANOVA indicated no statistically significant difference among the groups (F_6,2999_ = 1.63; *p*-value = 0.135). A comparison of mean Ct values of males (*n* = 2,422) and females (*n* = 584) revealed that males had a statistically significant higher mean Ct value (27.61 ± 5.20) than females (26.72 ± 5.31). The difference between the means of the two groups was −0.89 (95% C.I. = −1.36 to −0.42; *t*-test −3.71; df = 3,004; *p*-value < 0.001).

**Conclusion:** The study found no statistically significant difference in viral loads between age groups. It showed that females had a higher SARS-CoV-2 viral load compared to males. The findings have implications for preventive strategies. Further studies are needed to correlate viral load with clinical symptoms and outcomes.

## Introduction

The coronavirus disease 2019 (COVID-19), caused by the novel severe acute respiratory syndrome coronavirus-2 (SARS-CoV-2), is a global public health emergency. The mortality and morbidity caused by the disease constitute a major challenge to healthcare authorities around the globe. The challenge has been aggravated by lack of knowledge of the epidemiological and clinical attributes of the emerging disease (1).

On the 2nd of March 2020, a Saudi man at the Eastern Province coming from Iran confirmed positive for COVID-19 (2). Since then, the Saudi Ministry of Health (MOH) took the case seriously and isolated the patient and all of the contacts. Spreading of the virus was dramatically and several cases were reported in the same region due to the same reason of transmitting the infection of the first case. Such a disease creates huge burden on health care providers and governments as that of MERS Co-V in 2012 (3). Additionally, the worries about re-infection is of a major concern, whoever, protection from reinfection has been reported (4).

Age and sex are two important factors associated with risks and outcomes of COVID-19 disease (5). COVID-19 morbidity also seems to be affected by patient age and sex. It has been found that older age groups have more severe COVID-19 symptoms and higher fatality rates while children tend to have lower prevalence and milder symptoms than adults (6). Nonetheless, the potential role of asymptomatic or mildly symptomatic children in transmitting infection cannot be disregarded and is still debated (7–9). Preliminary evidence also indicated that sex has a role in the disease epidemiology. For example, a study from China indicated that men are at higher risk of severe disease and mortality compared to women (10).

Studying viral dynamics and their variation among population subgroups may help in understanding the role of age, sex, and other factors in the disease's epidemiology. One uncertainty of the new disease is COVID-19's viral dynamics and how they relate to factors in the population. Prior studies revealed that viral load was associated with disease severity and the number of days since the beginning of symptoms (11, 12). However, evidence on the association between viral load and other factors, including age and sex, has not been conclusive. Some studies found that higher viral load in the respiratory system was associated with higher in-hospital mortality and morbidity (13), and a higher risk of transmission (14); other studies found no such relationship (11). Understanding viral load dynamics and covariates is critical for identifying protective measures for individuals and the general public. Therefore, this study investigates the association of viral load with the age and sex of COVID-19 patients.

## Methods

### Study Design

The study reviewed electronic medical records of COVID-19 patients from Madinah city, Saudi Arabia. The study included all cases who tested positive between March 20 and May 22, 2020. Data were obtained from the Health Electronic Surveillance Network (HESN) database. HESN is a web-based platform run by the Ministry of Health to integrate public health programs in order to detect and control diseases, and monitor the population's health.

### Setting

Al-Madinah region has a population of 2.13 million. The main city in the region is Al-Madinah city, a holy city and home to the Prophet's Mosque. It attracts year-round visits from religious pilgrims from all over the world.

### Procedure Used by MOH in Specimens Collection of SARS-CoV-2 Patients

The MOH obligates all the health care workers who collect specimens to use appropriate personal protective equipment (PPE) such as, eye protection, surgical mask, while dealing with suspected Covid-19 patients. They must collect the respiratory specimen under aerosol generating procedure; personnel should wear a particulate proficient N95 respirator. Additionally, specimens should be placed for carriage in leak-proof specimen bags (secondary container) that have a detached sealable pocket for the specimen, with the patient's name tag on the specimen container (primary container). HESN printed lab requisitions must be sent with samples and national lab reception report and result values must be informed on HESN on their consistent time.

In terms of sources of sample testing, the most generally tested sources are nasopharynx and oropharynx (15). However, viral RNA in several biological specimens such as stool, tears and blood has been detected with variable positivity rates (16). Two types of samples are usually requested by the physicians be collected from the patients. First, lower respiratory tract samples, containing endotracheal aspirate, bronchoalveolar lavage fluid or sputum. Second, upper respiratory tract samples, nasopharyngeal swab (with or without oropharyngeal swab) in viral transport medium in a single tube. If initial testing is negative, repeat testing should be accomplished in case of there is a high index of suspicion. Finally, all results should be reported via HESN starting from registering of the case, for test requested select COVID-19, and select the designated regional laboratory. During shipment of samples they should be at 2–8°C and ship on ice pack to lab. Samples can be stored at 2–8°C for ≤48 h, if longer storage is required, samples should be stored at −70°C. If sample is frozen at −70°C, ship on dry ice https://www.moh.gov.sa/Ministry/MediaCenter/Publications/Documents/Coronavirus-Disease-2019-Guidelines-v1.2.pdf.

### Participants

All positive cases from Al-Madinah region were included in this analysis. Laboratory results of cases in the region were reported to the HESN database by the Al-Madinah Regional Lab. Epidemiological information on cases and samples originated from hospitals and primary healthcare centers in Al-Madinah region. In addition, samples collected during contact tracing or active surveillance were included in the HESN database.

### Variables

The outcome was the cycle threshold (Ct) value as measured by quantitative reverse transcriptase polymerase chain reaction (RT-PCR) assay. Lower Ct values indicate higher viral load and vice versa. Positive cases were defined as cases having Ct value of <35 in their sample. Age was calculated based on the date of birth and the sample submission date. Age was divided into seven categories (under 10 years old, 10 – <20, 20 – <30, 30 – <40, 40 – <50, 50 – <70, and 70 years or older).

### Statistical Analyses

Statistical analyses were performed using Stata 14.0 (StataCorp LLC, TX, USA). Data were presented as mean, standard deviation (SD), 95% confidence intervals (C.I.) or proportions as appropriate. One-way ANOVA was used to compare the differences of means between groups in a univariate analysis. Two-way ANOVA was used in a multivariable analysis to model the relationship between Ct values (outcome), and age group and sex (independent variables). Interaction was tested in the model. Equality of variances was assessed using Levene's Test.

### Ethical Considerations

Data collection was required by MOH as a part of the public health surveillance system. This investigation was conducted according to international and national ethical guidelines and approved by the regional research ethics committee of the Madinah Health Directorate (**IRB** number H-03-M-084).

## Results

This study used data from the national HESN database. It included 3,006 positive COVID-19 cases reported in the Al-Madinah region from March 20 to May 22, 2020. Approximately 80% of the study sample were males and half were in the 30 to 40-year-old age group (Table 1). The Ct value of the whole sample ranged from 15.08 to 35, with a mean of 27.44 (SD: 5.23; 95% C.I. = 27.25–27.66; Table 2). The means of Ct values varied between age groups from 27.05 to 27.82.

**Table 1:** Baseline characteristics of the sample by age and sex.

	**Total**	**Female**		**Male**	
**Age group**		**No**.	**%**	**No**.	**%**
<10	212	97	45.75	115	54.25
10 – <20	269	118	43.87	151	56.13
20 – <30	533	96	18.01	437	81.99
30 – <40	1,504	195	12.97	1,309	87.03
40 – <50	374	53	14.17	321	85.83
50 – <70	27	1	3.7	26	96.3
≥70	87	24	27.59	63	72.41
Total	3,006	584	19.43	2,422	80.57

**Table 2:** Summary statistics for Ct values in the study sample by sex and age group.

	***N***	**Mean**	**SD**	**SE**	**95% confidence interval**		*p*-value^*^
Overall	3,006	27.44	5.233	0.095	27.25	27.62	
**Sex**							
Males	2,422	27.61	5.200	0.106	27.40	27.82	<0.001
Females	584	26.72	5.312	0.220	26.29	27.15	
**Age group**							
<10	212	27.07	5.431	0.373	26.34	27.80	0.135
10 – <20	269	26.64	5.383	0.328	26.00	27.28	
20 – <30	533	27.37	5.269	0.228	26.93	27.82	
30 – <40	1,504	27.59	5.211	0.134	27.33	27.85	
40 – <50	374	27.63	5.047	0.261	27.11	28.14	
50 – <70	27	27.05	5.319	1.024	25.05	29.06	
≥70	87	27.82	5.059	0.542	26.75	28.88	

* *calculated by t-test for sex and one-way ANOVA for age groups*.

### Univariate Analysis

Analysis of the mean differences between age groups using one-way ANOVA indicated no statistically significant difference among the groups (F_6,2999_ = 1.63; *p*-value = 0.135).

A comparison of mean Ct values of males (*n* = 2,422) and females (*n* = 584) revealed that males had a statistically significant higher mean Ct value (27.61 ± 5.20) than females (26.72 ± 5.31). The difference between the means of the two groups was −0.89 (95% C.I. = −1.36 to −0.42; *t*-test −3.71; df = 3,004; *p*-value < 0.001).

### Multivariable Analysis

Two-way ANOVA analysis was conducted to examine the effect of age and sex on Ct value. There was no significant interaction between age and sex and Ct value (*F* = 1.61, *p*-value = 0.139). The main effect showed that the statistically significant difference between males and females persisted after adjustment for age group (*p*-value = 0.002). The two-way ANOVA analysis also showed no statistically significant difference between age groups (Table 3).

**Table 3:** Two-way Analysis of Variance of Ct values with age groups and sex.

**Source**	**Partial SS**	**df**	**Mean Square**	***F***	***p*-value**
Model	524.575	7	74.939	2.750	0.008
Age group	150.112	6	25.019	0.920	0.481
Sex	257.015	1	257.015	9.430	0.002
Residual	81, 752.071	2,998	27.269		

## Discussion

This study compared COVID-19 viral load, as indicated by Ct value, across seven age groups, and between men and women. It found that viral load in patients did not differ by age group, but was higher among women than men. The argument in this study was that viral load is proportional to infectiousness of viral infections. The relationship between viral load and risk of transmission has been established in other viral diseases (17, 18); COVID-19 seemed likely to follow a similar pattern (19). Therefore, identifying factors related to viral load could aid prevention strategies and identification of groups contributing to higher transmission risk.

The distribution of viral load observed in this study is consistent with results from previous studies. In Switzerland, Jacot et al. (20) analyzed data on 4,172 positive patients and concluded there was no statistically significant difference between 5 year age groups. Another study from Switzerland compared 352 patients older than 16 years with 53 children under 16 years old and found a similar mean viral load between the two groups (21). Similarly, a study in the United States which included 4,428 patients with positive lab results found no variation in mean and median viral load values (22). Notably, other studies with smaller sample sizes had conflicting results regarding the relationship between SARS-CoV-2 viral load and age. One study of 23 patients concluded that older age groups had higher viral loads (23); another study (24) of 145 patients found that children under 5 years of age had higher viral loads.

This study found higher SARS-CoV-2 viral loads (lower Ct values) among females compared to males. Previous studies were not conclusive on the sex difference in viral load. Jacot et al. (20) and Kleiboeker et al. (22) reported comparable viral loads between males and females. Takahashi et al. (25) found the clinical status of patients was a modifier for the relationship between sex and viral load. Finding that sex effects viral load and immunological response to infectious disease is not surprising; it has been demonstrated in other diseases. This is thought to be related to a difference in immune response in which females develop a higher immune response to infectious agents, making them less susceptible to diseases (26). Gender differences in the response to hepatitis B virus were reported in humans as well (27). Similarly, sex difference seems to play a role in COVID-19 infection; various mechanisms have been suggested to explain this difference (28). Women mount stronger immune responses to infections as well as vaccinations and outlive men (29). As we do not have enough clinical data to investigate the disease severity and correlate that with age and gender, other study showed that men tended to get much sever cases than women. Additionally, older age was greater number in the deceased patients than in the patients who survived. However, several reports showed that there was no difference in terms of susceptibility to SARS-CoV-2 between women and men (10).

The present study provides evidence on age and sex differences in SARS-CoV-2 viral load in a large sample size. It also included a good number of young children who are often less represented in similar studies.

Limitations of the study are a lack of clinical data and the consequent inability to correlate laboratory values with illness stage or severity. Additionally, only respiratory tract specimens were considered and the study non-including alternative shedding routes and that could represent future developments of the study.

In conclusion, the study found no statistically significant difference in viral loads between age groups. It showed that females had a higher SARS-CoV-2 viral load compared to males. The findings have implications for preventive strategies. Further studies are needed to correlate viral load with clinical symptoms and outcomes.

## Data Availability Statement

The raw data supporting the conclusions of this article will be made available by the authors, without undue reservation.

## Ethics Statement

The studies involving human participants were reviewed and approved by Madinah Health Directorate (IRB number H-03-M-084). Written informed consent to participate in this study was provided by the participants' legal guardian/next of kin.

## Author Contributions

AA-Z and WM conceived and designed the study, conducted the preliminary review of articles, wrote the initial and final drafts of the article, and provided logistic support. AA-Z, WM, and AA provided research scope, and collected and organized the extracted data. AA-Z, WM, AD, and HA analyzed and interpreted the data, have critically reviewed and approved the final draft and are responsible for the content and similarity index of the manuscript. All authors contributed to the article and approved the submitted version.

## Conflict of Interest

The authors declare that the research was conducted in the absence of any commercial or financial relationships that could be construed as a potential conflict of interest.
